# Visual-Based Children and Pet Rescue from Suffocation and Incidence of Hyperthermia Death in Enclosed Vehicles

**DOI:** 10.3390/s23167025

**Published:** 2023-08-08

**Authors:** Mona M. Moussa, Rasha Shoitan, Young-Im Cho, Mohamed S. Abdallah

**Affiliations:** 1 Computer and Systems Department, Electronics Research Institute (ERI), Cairo 11843, Egypt; mona_moussa@eri.sci.eg (M.M.M.); rasha.shoitan@eri.sci.eg (R.S.); 2 Department of Computer Engineering, Gachon University, 1342 Seongnam-daero, Sujeong-gu, Seongnam 13415, Republic of Korea; 3 Informatics Department, Electronics Research Institute (ERI), Cairo 11843, Egypt; 4 AI Laboratory, DeltaX Co., Ltd., 3F, 24 Namdaemun-ro 9-gil, Jung-gu, Seoul 04522, Republic of Korea

**Keywords:** in-cabin monitoring, child in vehicle, hyperthermia, NanoDet, YOLO

## Abstract

Over the past several years, many children have died from suffocation due to being left inside a closed vehicle on a sunny day. Various vehicle manufacturers have proposed a variety of technologies to locate an unattended child in a vehicle, including pressure sensors, passive infrared motion sensors, temperature sensors, and microwave sensors. However, these methods have not yet reliably located forgotten children in the vehicle. Recently, visual-based methods have taken the attention of manufacturers after the emergence of deep learning technology. However, the existing methods focus only on the forgotten child and neglect a forgotten pet. Furthermore, their systems only detect the presence of a child in the car with or without their parents. Therefore, this research introduces a visual-based framework to reduce hyperthermia deaths in enclosed vehicles. This visual-based system detects objects inside a vehicle; if the child or pet are without an adult, a notification is sent to the parents. First, a dataset is constructed for vehicle interiors containing children, pets, and adults. The proposed dataset is collected from different online sources, considering varying illumination, skin color, pet type, clothes, and car brands for guaranteed model robustness. Second, blurring, sharpening, brightness, contrast, noise, perspective transform, and fog effect augmentation algorithms are applied to these images to increase the training data. The augmented images are annotated with three classes: child, pet, and adult. This research concentrates on fine-tuning different state-of-the-art real-time detection models to detect objects inside the vehicle: NanoDet, YOLOv6_1, YOLOv6_3, and YOLO7. The simulation results demonstrate that YOLOv6_1 presents significant values with 96% recall, 95% precision, and 95% F1.

## 1. Introduction

In recent years, car manufacturers have significantly invested in increasing vehicle and passenger safety via monitoring and recognizing human actions inside and outside a vehicle. Some of these investments are concentrated on monitoring the driver’s behavior, including distraction and drowsiness, to increase their ability to control the vehicle and reduce road accidents. Driver distraction is detected by examining the driver’s attention to the driving process and alerting them in scenarios of attention loss [1,2,3,4,5]. Drowsiness is detected by monitoring the facial expression of the driver based on computer-vision-based techniques [6,7,8,9,10,11] or by measuring the physiological signals of the driver using an electrocardiogram (ECG) [12] and heart rate monitoring [13]. Most driver-behavior-monitoring methods have been designed to reduce crash-related accidents by addressing driver behavior. However, there are still non-crash-related accidents, such as the death of a child inside a closed vehicle due to hyperthermia. The National Highway Traffic Safety Administration (NHTSA) has revealed that, since 1998, over 900 children under 14 have died from heatstroke because they unintentionally locked themselves inside a car or are intentionally left in vehicles when parents leave to undertake quick tasks such as shopping. Research shows that on a sunny day with ambient temperatures of 20 °C, the internal vehicle temperature reaches 40 °C within the first half hour of being turned off, and then continues to increase even further, exceeding the temperature necessary for the human body to sustain equilibrium. With the increasing problem of heat-related child deaths, researchers’ and vehicle manufacturers’ attention have been drawn to developing various solutions for detecting forgotten children inside a car and alerting their parents. These solutions can be categorized into sensor-based methods and computer-vision-based methods. In sensor-based methods, different sensors, such as motion sensors, temperature sensors, microwave sensors, and pressure sensors, are embedded inside the vehicle to detect the presence of a child [14,15]. Nevertheless, these methods have not been reliably able to detect children who are left behind in a car. In computer-vision-based methods, deep learning models are integrated with camera sensors to detect the existence of a child in a car [16,17,18]. These methods focus only on forgotten children, whereas some people forget pets inside their car. Moreover, these methods only detect the presence of a child, irrespective of whether this child is with their parents or alone, which can yield a false alarm.

This research introduces a visual-based method to rescue children and animals forgotten in enclosed vehicles from suffocation. The proposed research creates a dataset for children, pets, and adults inside an enclosed vehicle. This dataset is collected from the web, considering different angles, sizes, illumination conditions, pet types, clothes, and skin colors. Since the occlusion and small size of children and pets are critical challenges in this task, the dataset also covers these issues. Different state-of-the-art detection algorithms are fine-tuned on the custom dataset for detecting children, pets, and adults. When the system detects a child or pet in the car without an adult, the system sends a notification alarm to the parents. The main contributions of this research paper are as follows:

1. This research presents a system to rescue children and animals forgotten in enclosed vehicles from hyperthermia death based on a visual-based solution.
2. A new dataset is constructed containing 4957 images divided into three classes: adults, children, and animals. Also, the scenes contain real objects, with neither synthetic datasets nor toy objects.
3. Fine-tuning is performed on four deep learning models to detect children, pets, and adults: YOLOv6_1, YOLOv6_3, YOLOv7, and NanoDet.

## 2. Related Work

The development of different technologies for detecting children in enclosed vehicles has commenced on several fronts in response to the growing issue of hyperthermia-related deaths in children. Developing a reliable detection system that is able to detect children and pets in enclosed vehicles is a very challenging task. This section presents a variety of solutions to overcome this issue. NHTSA compares different systems for detecting a forgotten child in a vehicle, such as systems that test the presence or absence of a child in the Child Restraint System (CRS) based on pressure sensors. The drawback of these systems is that they cannot discriminate between heavy items and a child in a seat. Therefore, it sends false alerts to the car owner. Another system adds a switch in the chest clip to decide whether the chest clip is closed and the child is seated in the CRS [19]. However, these systems cannot detect pets or children not seated in the CRS. David Chua et al. [14] integrated passive infrared (PIR) motion sensors, ultrasonic sensors, carbon dioxide sensors, and infrared temperature sensors in a system to overcome the shortage of using only one sensor. Hashim et al. [15] designed a system based on the Global System for Mobile Communication (GSM) and a microcontroller to recognize any movement made by children who were left behind in a car, as well as sound or voice. Norizam Sulaiman et al. [20] introduced a system that recognizes the signal generated from motion, odor, temperature, and voice and sends a Short Messaging System (SMS) to the parents, turns on the car horn, and lowers the car window to let the toxic gas out. Dian Shi et al. [21] introduced a system that detects a child or pet in the rear seat using static channel state information of WiFi signals. It exploits the fact that dissimilar objects lead to differences in the reflected channel state information. Yixuan Chen et al. [22] introduced a system to detect in-vehicle passengers based on frequency-modulated continuous wave (FMCW) radar. In this system, point cloud clustering, postclustering processing, and a state machine determination algorithm are integrated to solve the instability produced by passengers’ sitting poses and significant movements in a car.

Machine learning and deep learning have recently demonstrated promise for image classification and detection; thus, different vehicle manufacturers and researchers apply them to in-vehicle child detection. Henrik Hallberg [16] developed a Raspberry Pi and camera system to classify real-time images based on convolutional neural networks to detect children inside vehicles. The system was trained on images collected from the web and real images captured by the authors of a doll inside a car seat. Yusen Hu [17] proposed a multitask cascaded convolutional neural network for detecting the presence of a child. The system was trained on a constructed child-in-vehicle dataset for children younger than seven years. These visual-based methods concentrate on forgotten children; however, some people forget that their pets suffocate inside vehicles. Asha Banu et al. [18] developed a visual-based system designed from an Arduino Pro Mini board equipped with an IR proximity sensor to detect whether a child has been left in a car. A dataset was created from eight individual children. The haar features and Local Binary Pattern Histogram (LBPH) [23] were presented for feature extraction and face recognition.

To the authors’ knowledge, these are the only visual-based methods introduced for detecting forgotten children in enclosed vehicles. The abovementioned visual-based methods classify the images into two classes: zero or one. Zero means the image does not include a child, while one means that the image includes a child, and a notification is sent to the parents. However, sometimes an adult exists in the car with the child, and in this case, a false notification is sent. Therefore, several models are introduced and categorized into two-stage and single-stage methods for multiple object detection and localization in the same image. Two-stage methods include faster R-CNN [24] and Mask R-CNN [25], while single-stage methods include YOLO [26,27,28,29,30], SSD [31], and Nanodet [32]. Two-stage algorithms are more accurate at detecting objects than single-stage methods. However, these methods are unsuitable for embedded systems because they require high processing time and memory. This has encouraged researchers to improve the quality of these methods and design new models that are accurate and lighter than YOLO6 v1 [28], YOLO6 v2 [27], YOLO7 [29], and Nanodet.

Based on these related works and the aim of this research paper to implement the system on Raspberry Pi, it was decided to fine-tune YOLO6 v1, YOLO6 v2, YOLO7, and Nanodet on the proposed dataset to select the best model for the proposed framework.

## 3. The Proposed Framework

A family’s worst scenario comes true when a child or pet is unintentionally left in a car because heat builds up fast on a sunny day, leading to the child’s or pet’s death. Reducing the number of fatalities in cars has become a mission-critical concern for manufacturers. This research presents a technological solution that can prevent these mishaps by detecting the presence of children and pets inside a vehicle via a visual-based solution. The suggested children and pet presence detection system follows the monitoring-by-detection paradigm. First, a dataset was created for children, pets, and adults from different online sources, and then, a combination of cutting-edge models like YOLOv6_1, YOLOv6_3, YOLOv7, and NanoDet was fine-tuned on this dataset. This work aimed to operate (transplant) the visual-based system on embedded or onboard devices. Therefore, these models were selected because they are the most often used detection frameworks in industrial applications due to their great speed–accuracy balance.

The child and pet detection system uses a monitoring camera (a middle mirror camera) to detect the existence of an infant or pet left in a car and notify the driver. The system workflow is summarized in the following steps:

1. When a car arrives at its intended location and the driver gets out of the car, the proposed system waits a little while before the camera sensor is activated to capture and detect any possible children or pet that might be there.
2. The proposed system applies an object detection model to detect the objects in the image.
3. The proposed algorithm checks if an adult is in the car based on the detected objects. If an adult exists, the proposed algorithm checks whether there is a child or a pet with the adult and stops or waits for a while to investigate if the adult gets out of the car.
4. If a child or pet exists without an adult, the car may carry out several steps, such as sounding the horn and flashing the warning lights, preventing the windows and doors from locking, and rechecking to be sure that the parents have taken the child or the pet with them.

**Figure 1:**
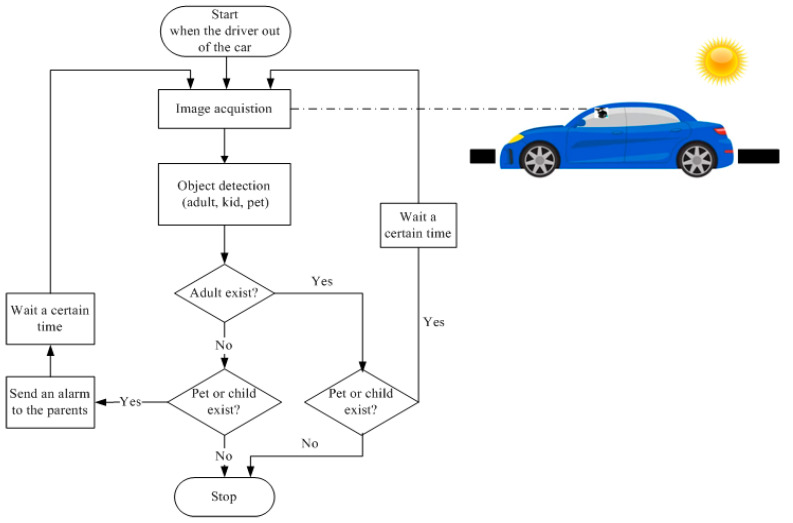
The flowchart of the suggested visual-based child and pet in vehicle detection system.

Figure 1 shows a flowchart of the suggested visual-based system, which includes a camera that can perform various in-cabin monitoring tasks. The pros of the suggested system are that cameras can be a cost-effective technique to find a child in a car, especially if the camera is already in the vehicle’s interior for other purposes, even though there are restrictions due to line-of-sight barriers. Moreover, the suggested system, which is the most thorough method for detecting the presence of children, may detect a child or pet through other obstructions like blankets or toys due to the high level of accuracy of the suggested detection models. Moreover, the system strives to minimize false positives to prevent users from dismissing any ensuing alert. Because the system’s accuracy mainly depends on object detection models, the following subsections briefly describe these models and their architectures.

### 3.1. YOLOv6_1

YOLOv6_1′s architecture consists of three main parts: the backbone, neck, and head [28]. The backbone is the feature extractor to extract deep-level features of images. Although the backbone largely influences how features are represented effectively, its structure also substantially impacts how well inferences perform. In order to effectively use the hardware architecture’s processing capability and decrease inference latency, EfficientRep was created as the backbone of YOLOv6_1. The EfficientRep network was designed based on the RepVGG structure [33] to use the advantages of multibranch topology during the training phase and increase classification efficiency. The neck is the feature aggregator that combines data from layers at different levels. To create a pyramid feature map, Rep-PAN is used as the neck network to combine low-level and high-level semantic characteristics. The head, made up of convolutional layers, is supplied with aggregated information to predict the locations and classes of objects. Furthermore, the training strategies of YOLOv6_1 are improved over the conventional YOLO to increase its detection accuracy by using an anchor-free paradigm, SimOTA [34], as a label assignment method, and SIoU [35] and GIoU [36] as bounding box regression loss functions. YOLOv6_1 increases the latency, reduces the delay, preserves the accuracy, and decreases the utilization of the memory bandwidth of the GPU compared to conventional YOLO versions.

### 3.2. YOLOv6_3

YOLOv6_3 is an upgraded version of YOLOv6_1 in terms of the network architecture and training strategies, enabling it to attain the highest level of accuracy for real-time object detection [27]. In YOLOv6_3, a bi-directional concatenation (BiC) module is added to the Rep-PAN network to fuse an additional low-level feature from the backbone into the neck for preserving more precise localization signals, which are essential for localizing small objects. Moreover, anchor-aided training (AAT) is introduced to benefit from the pros of anchor-based and anchor-free paradigms without affecting inference performance.

### 3.3. YOLOv7

The network architecture of YOLOv7 follows the architecture of the conventional YOLO series [29]. YOLOv7 introduces a pair of architecture modifications and a series of bag-of-freebies to improve the accuracy with the same inference speed, only increasing the training time. In the backbone network, the extended efficient layer aggregation network (E-ELAN) is introduced to help the network to learn more features. The second, YOLOv7, develops compound model scaling for a concatenation-based model in which the block depth and width are scaled with the same factor to reduce the hardware usage of the model. The bag-of-freebies introduced in YOLOv7 is planned reparametrized convolution that uses RepConv without identity connection (RepConvN) and coarse label assignment for auxiliary head and fine label assignment for the lead head.

### 3.4. NanoDet

NanoDet is a single-stage anchor-free object detection model inspired by the FCOS style (a fully convolutional anchor-free detector) [32]. The architecture of NanoDet consists of three parts: the feature extraction network (backbone), the feature fusion network (neck), and the prediction head. ShuffleNet is used as the backbone network. PAFPN is used for feature fusion [37] to merge deep and low features. Quality focal loss (QFL) [38], focal distribution loss (DFL) [39], and GIoU loss are combined to compose the loss function for evaluating the head output. Moreover, an auxiliary assign guidance module (AGM) is added during the training to improve the model performance.

## 4. Evaluation Performance

### 4.1. Data Collection

**Figure 2:**
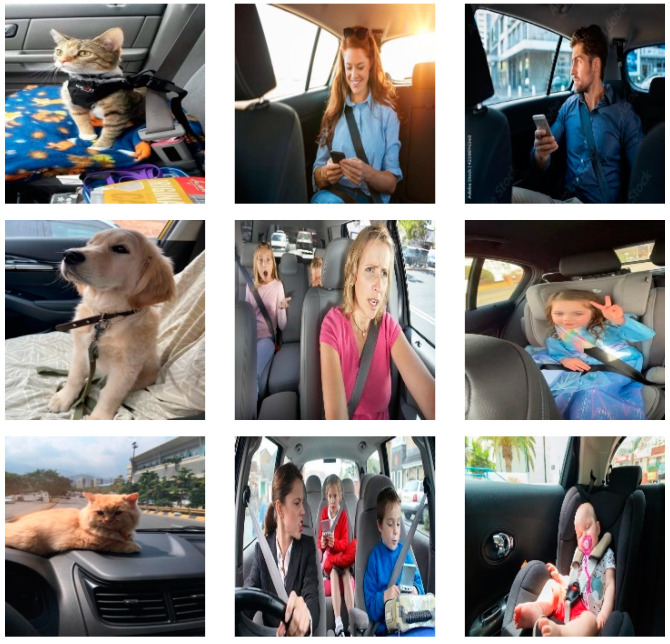
Sample images from the proposed dataset.

Real images were collected from various online sources such as Google, Pinterest, and Flickr for training the models. The downloaded images included adults, children, and pets inside vehicles. The authors considered that the images should vary in illumination, skin color, pet type, clothes, and car brand to increase the model’s robustness. A sample from the images is shown in Figure 2. Since training detection models generally demand many images, various augmentation algorithms are applied to the training images to reach sufficient image amounts. These augmentation algorithms are blurring, sharpening, brightness, contrast, and noise (additive Laplace the Noise (scale = 20), perspective transform, and fog effect). The number of images after the augmentation process was 3509, and these images were annotated using the YOLO BBox Annotation Tool (Ybat) [40]. The total number of objects and the number of objects per class in these images were counted, and their numbers are tabulated in Table 1. These objects were divided into 70% training, 15% testing, and 15% validation, considering no overlap between the objects to avoid biased results.

### 4.2. Training Details and Evaluation Index

YOLOv6_1, YOLOv6_3, YOLO7, and NanoDet were implemented in PyTorch. YOLO6 and YOLO7 were trained with an SGD optimizer. YOLOv6 used a cosine learning rate scheduler, while YOLO7 used a linear one. The learning rate for YOLO 6 started at lr:0.0032 and reached lr:0.12. For YOLO7, it started at lr:0.01 and reached lr:0.1. The learning rate for NanoDet started at lr:1.00 × 10^−7^ and reached lr:5.00 × 10^−5^. All the detection models were trained for 300 epochs with an image size of 640 × 640. All experiments were conducted based on an 11th Gen Intel(R) Core (TM) i7-11800H with 32 GB of RAM NVIDIA GeForce RTX 3060.

The performance of NanoDet, YOLOv6_1, YOLOv6_3, and YOLO7 was evaluated using recall, precision, F1-score, and IoU. These metrics were calculated according to the following formulas:

$$precison=\frac{TP}{TP+FP}$$

$$Recall=\frac{TP}{TP+FN}$$

where TP, TN, FN, and FP are the number of true positives, true negatives, false negatives, and false positives, respectively. Precision measures how the ratio of objects is correctly predicted. Recall is the accuracy ratio of objects predicted and how they match to categories. These indices must be as high as possible.

## 5. Simulation Results

**Figure 3:**
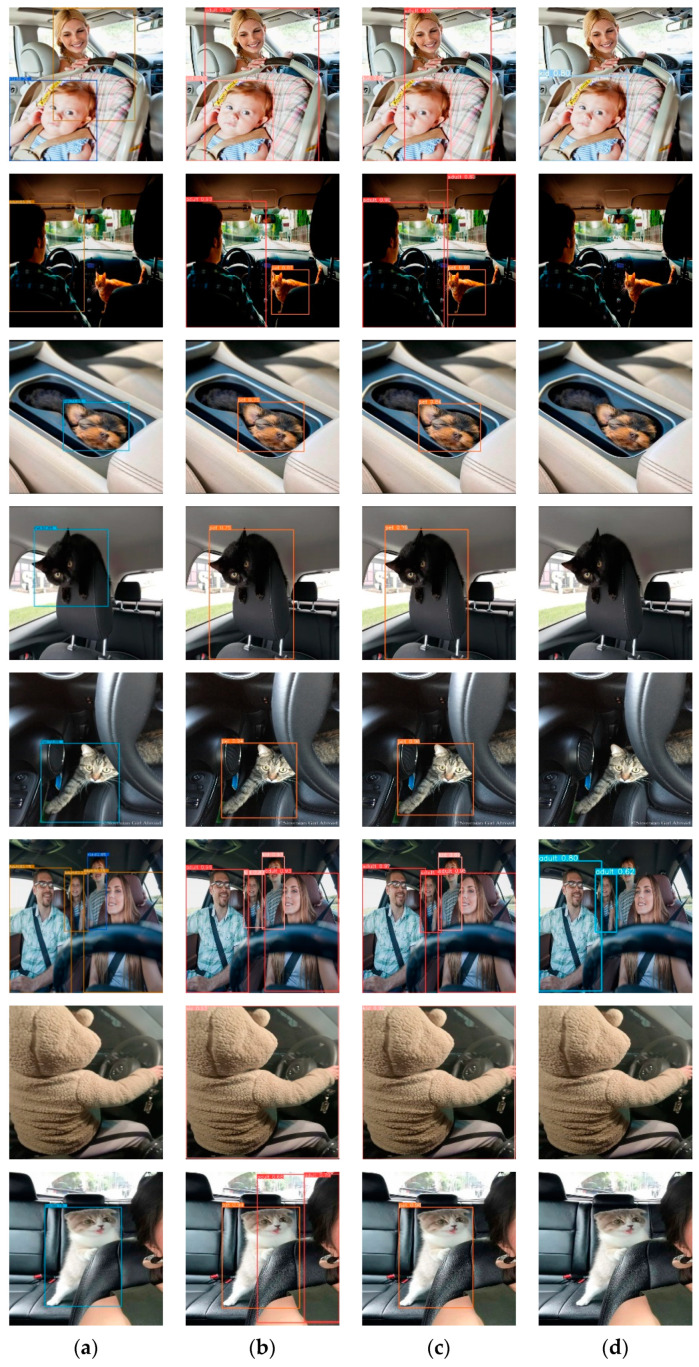
The child- and pet-in-vehicle detection results for each model: (**a**) NanoDet; (**b**) YOLO6 v1; (**c**) YOLO6 v3; (**d**) YOLO7.

This section demonstrates the performance analysis of the fine-tuned detection models NanoDet, YOLO6 v1, YOLO6 v3, and YOLO7 for detecting forgotten children and pets in vehicles. All the mentioned models were trained on the COCO dataset and fine-tuned on the proposed dataset. Table 2 compares these models regarding recall, precision, F1, IoU, and model size. It can be observed from the table results that YOLO6 v1 had the highest recall, F1, and IoU compared to the other detection methods. On the other hand, it can be noticed that the size of the NanoDet model was approximately half that of YOLO6 v1 and YOLO v3. However, its recall and IoU values were smaller than YOLO6 v1 by approximately 5, reflecting that NanoDet could not correctly identify some objects, and the detected bounding boxes were not matched with the ground truth. The selected common image samples for the detection models in Figure 3 demonstrate these results. It can be seen in the figure that YOLO6 detected all the objects in the selected images, while YOLO7 failed to detect most of them. Moreover, NanoDet performed as well as YOLO6 in some images but failed to detect others, such as the baby image in row 7.

**Figure 4:**
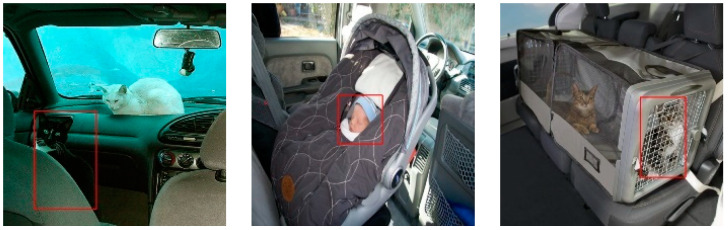
Common missed detections in the detection models.

By comparing the ground truth with the detected objects, it was found that all the detection models failed to detect the objects marked with a red bounding box, as shown in Figure 4. It can be noticed from the figure that the cat in the first image is so dark that the human eye is not able to distinguish it from the background. In the second image, the child’s face is so tiny that none of the models can detect it. The cat in image three is behind the gates of the pet box and just visible enough to be detected.

**Figure 5:**
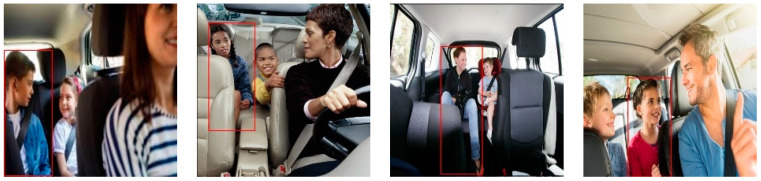
Common false classifications in the detection models.

Furthermore, it was found that all the detected algorithms could not classify the objects surrounded by red boxes in the images in Figure 5. In images one, two, and four, all the models misclassified the children and detected them as adults, while the adult in image three was detected as a child. This issue arose because teenagers can be misclassified as adults or as children.

**Figure 6:**
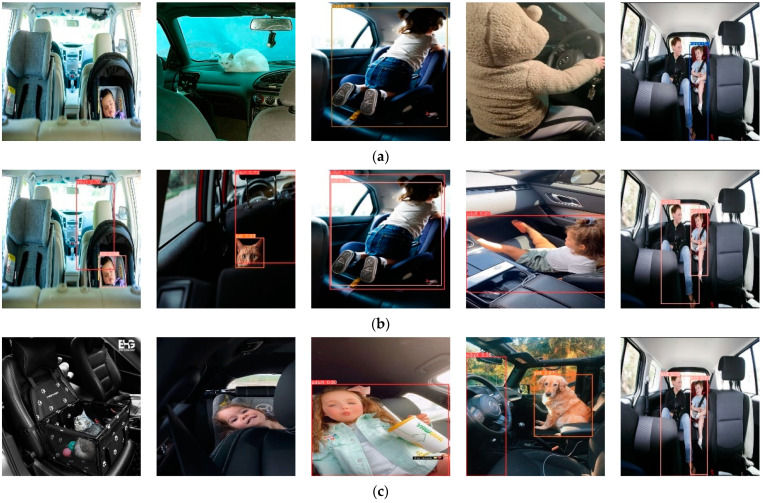
False-safe and false-dangerous examples: (**a**) NanoDet; (**b**) YOLO6 v1; (**c**) YOLO6 v3.

As mentioned, the best detection model will be deployed on an embedded system to finalize a product for the market. Therefore, the detection models were evaluated to investigate the number of images in which the system fails to determine whether an adult exists with a child or not. These images were divided into two groups: false safe and false dangerous. False safe refers to images with only a child or a pet inside the vehicle and which are not detected or misclassified by the models. Also, false safe refers to images in which the models detect an adult with a child or pet, but the adult does not exist in the car. In this case, the system will not notify the car owner, and the child will be in danger. False dangerous refers to the images where the models detect and classify the objects as a child or a pet without an adult, but no child or pet is inside the car. In this case, the system will send the car owner the wrong notification: “You forgot a child in the car”. Table 3 presents the number of images for each model in cases of false safe or false dangerous. It can be perceived from the table that the NanoDet model produced the highest number of false images, while YOLO6 v3 produces the lowest number of false images. Figure 6 shows examples of false-safe and false-danger cases. The first four columns represent false safe, and the last represents false dangerous. It can be observed from the figure that false-safe results were due to the models not being able to detect a child or pet, as in the images in column 1 row 1, column 1 row 3, and column 4 row 1. Moreover, the models detected an adult in the car with a child or a pet while there was no adult, as in the images in column 2 row 2, column 3 row 2, and column 4 row 3. For the false-danger images in the last columns in the figure, it can be remarked that NanoDet detected the child and did not detect the adult; thus, the system would send a false notification that the child is alone in the car. On the other hand, YOLO6 v1 and YOLO6 v2 detected the adult as a child. So, the system would consider the car to have two children and notify the owner.

**Figure 7:**
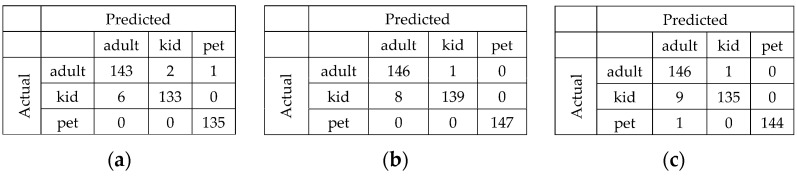
Confusion matrix for the detection models: (**a**) NanoDet; (**b**) YOLO6 v1; (**c**) YOLO6v3.

From the previous results, the models that had the best performance were NanoDet, YOLO6 v1, and YOLO6 v3. Therefore, the confusion matrices for these models are presented in figures for a detailed comparison. Moreover, the precision, recall, and F1 for each class of the three models are introduced in Figure 7 to clarify the confusion matrix results. It can be perceived from the confusion matrices that the child class was the most misclassified among the three models. The reason is that some children in their teens look like adults.

**Figure 8:**
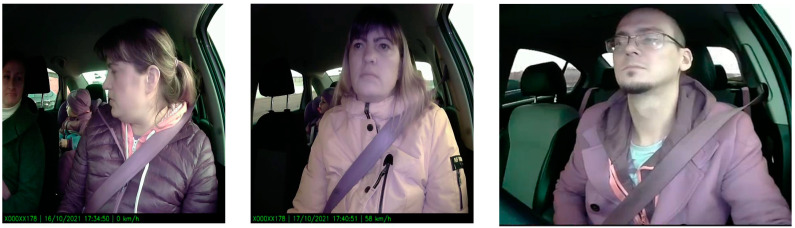
Sample frames from the DriverMVT dataset.

Moreover, the fine-tuned algorithms were evaluated on the DriverMVT (Driver Monitoring with Videos and Telemetry) dataset [3] to generalize the model. However, this dataset presents driver behavior inside a vehicle, like drowsiness and an unbuttoned belt. Therefore, the recorded videos were watched, and the frames with adults and children were extracted and annotated to evaluate the fine-tuned algorithm. Figure 8 presents samples from these frames. It can be noticed from the figure that the dataset suffers from insufficient illumination, which is considered a challenge for fine-tuned algorithms. Table 4 compares the best detection models regarding recall, precision, and F1. It can be observed from the table results that YOLOv6_1 outperformed the other detection models on the DriverMVT dataset.

**Figure 9:**
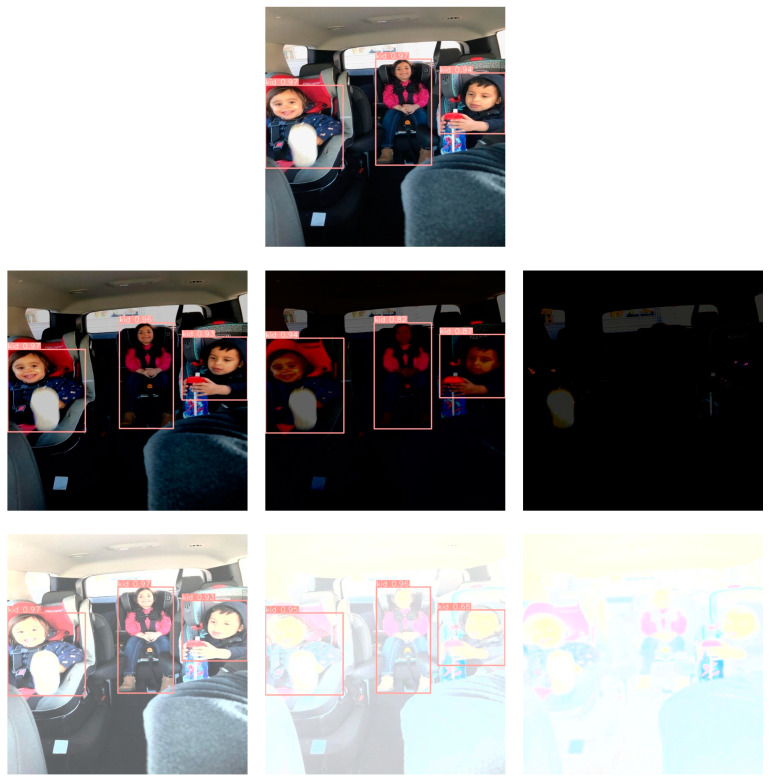
Effect of varying the illumination degree. First row: original image. Second row: decreasing illumination. Third row: increasing illumination.

Figure 9 proves the high robustness of the proposed system against the variation in illumination degree. The first row of the figure shows the original image and the detected objects in it, while the second row demonstrates the detection efficiency under low illumination; as can be noticed, the detection is accurate even in dim lighting. The third row of the figure shows the detection efficiency under increased illumination, and the system showed high accuracy, even in dazzling lighting.

## 6. Conclusions

This research paper introduced a visual-based solution for rescuing children and pets from hyperthermia death in enclosed vehicles. An image dataset was constructed from the web for the in-cabin monitoring of vehicle occupants, including children, pets, and adults, considering variations in lighting, skin tone, pet breed, clothing, and automobile brand to ensure model robustness. The collected images were augmented based on different algorithms to increase the number of images for better training performance. YOLO6 v1, YOLO6 v2, YOLO7, and NanonDet were selected as object detection models to be evaluated in this research because of their high level of accuracy for real-time object detection, which suits industrial systems. These detection models were fine-tuned on the proposed dataset, and YOLO6 v1 yielded the best results in detection accuracy. The system starts when a car pulls up to its destination and the driver exits; a camera sensor is then activated after a brief delay to catch and detect any children or pets that may be there. The fine-tuned object detection model is applied to the captured image to detect children, pets, or adults. If there is a child or pet without an adult in the vehicle, the system sends many notifications to the parents until they collect the child or pet.

## Figures and Tables

**Table 1:** Dataset description.

	No. of Images	No. of Objects	No. of Objects/Class	
**Training set**	2850	4057	adults	1413
			child	1347
			pets	1297
**Test set**	325	450	adults	150
			child	150
			pets	150
**Validation set**	334	450	adults	150
			child	150
			pets	150

**Table 2:** Performance analysis of the fine-tuned detection models: NanoDet, YOLO6 v1, YOLO6 v3, and YOLO7 in terms of recall, precision, F1, IoU, and model size.

	Recall	Precision	F1	IoU	Model Size
NanoDet	91.333	95.581	93.409	78.98	17.1 MB
YOLO6 v1	96.0	94.945	95.47	83.75	37.16 MB
YOLO6 v3	94.444	95.506	94.972	83.27	38.68 MB
YOLO7	48.667	98.206	65.082	40.34	73.079 MB

**Table 3:** The number of false-safe and false-dangerous cases for each model.

	False Safe	False Dangerous
NanoDet	9	6
YOLO6 v1	7	1
YOLO6 v3	5	2

**Table 4:** The performance of the fine-tuned models (NanoDet, YOLO6 v1, YOLO6 v3, and YOLO7) on the DriverMVT dataset in terms of recall, precision, F1, and IoU.

	Recall	Precision	F1	IoU
NanoDet	65.96	93.94	77.5	59.14
YOLO6 v1	97.87	100	98.93	84.15
YOLO6 v3	93.617	100	96.703	80.943

## Data Availability

Not applicable.
